# Infant attachment predicts bodily freezing in adolescence: evidence from a prospective longitudinal study

**DOI:** 10.3389/fnbeh.2015.00263

**Published:** 2015-10-12

**Authors:** Hannah C. M. Niermann, Verena Ly, Sanny Smeekens, Bernd Figner, J. Marianne Riksen-Walraven, Karin Roelofs

**Affiliations:** ^1^Behavioural Science Institute (BSI), Radboud UniversityNetherlands; ^2^Donders Institute for Brain, Cognition and Behaviour, Radboud UniversityNetherlands; ^3^Faculty of Psychology and Educational Sciences, Open University of the NetherlandsHeerlen, Netherlands

**Keywords:** freezing, adolescent, infant, attachment, longitudinal

## Abstract

Early life-stress, particularly maternal deprivation, is associated with long-lasting deviations in animals’ freezing responses. Given the relevance of freezing for stress-coping, translational research is needed to examine the relation between insecure infant-parent attachment and bodily freezing-like behavior in humans. Therefore, we investigated threat-related reductions in body sway (indicative of freezing-like behavior) in 14-year-old adolescents (*N* = 79), for whom attachment security was earlier assessed in *infancy*. As expected, insecure (vs. secure) attachment was associated with less body sway for angry vs. neutral faces. This effect remained when controlling for intermediate life events. These results suggest that the long-lasting effects of early negative caregiving experiences on the human stress and threat systems extend to the primary defensive reaction of freezing. Additionally, we replicated earlier work in adults, by observing a significant correlation (in adolescents assessed as securely attached) between subjective state anxiety and reduced body sway in response to angry vs. neutral faces. Together, this research opens venues to start exploring the role of freezing in the development of human psychopathology.

## Introduction

Freezing is a major defensive stress-response in animals. It is characterized by reduced body motion and is often accompanied by decreased heart rate (Fanselow, 1984; Schenberg et al., 1993). Upon encounter of threat, the parasympathically driven freeze-reaction facilitates fast risk assessment. It promotes hyper-vigilance for environmental cues that in turn optimizes subsequent sympathetically driven fight-or-flight actions (Lang et al., 1997). Freezing is therefore considered as generally adaptive and necessary for adequate stress coping. However, exacerbated freezing can also negatively influence the development and functioning of the individual (Bovin et al., 2008). For example, amplified freezing has been observed in animals and humans with a history of anxiety and traumatization (Sanders and Knoepfler, 2008; Qi et al., 2010; Roelofs et al., 2010; Hagenaars et al., 2012). In addition, it has been found to enhance the chance of later psychopathology (Bovin et al., 2008). Research in rodents and primates has shown that the experience of early life stress, such as postnatal separation from the animal’s mother or low levels of maternal licking and grooming in rats, can result in dramatic changes in these defensive stress-reactions. Early negative caregiving experiences have specifically been associated with exacerbated freezing, life-long anxious or avoidant behavior, and maladaptive stress-coping in rodents and primates (Harlow, 1962; Caldji et al., 2000; Menard et al., 2004; Sanders and Knoepfler, 2008; Lukkes et al., 2009; Haller et al., 2014). In humans less is known about these relations. The main goal of the present longitudinal investigation was to test prospectively whether infant-parent attachment is related to freezing behavior in humans during adolescence.

Longitudinal research in humans has identified insecure infant-parent attachment—associated with insensitive caregiving—as a potent risk factor for negative developmental outcomes, including emotional disorders such as anxiety and aggression (Renken et al., 1989; Warren et al., 1997; Burgess et al., 2003; Shamir-Essakow et al., 2005; Smeekens et al., 2007b; Colonnesi et al., 2011; Groh et al., 2012). Accordingly, the human early life stress model (Loman and Gunnar, 2010) predicts that insecure infant-parent attachment can potentially lead to increased defensive responses such as freezing in humans. Particularly in the first years of life, when the developing threat and stress systems are most plastic and open to modifications by experience, elevated levels of chronic stress, which are likely experienced by insecurely attached infants, may lead to an overly reactive stress-response system and a hypersensitive threat-appraisal system. In turn, this may bias the threat system to rapidly orchestrate exaggerated defensive behaviors, such as exaggerated freezing. In contrast, secure infant-parent attachment, reflecting a history of sensitive and responsive caregiving (Susman-Stillman et al., 1996), is thought to buffer against those amplified stress-reactions (Loman and Gunnar, 2010).

In light of the potential importance of maladaptive defensive freezing behavior for human psychopathology, new methods have been developed to objectively assess bodily freezing in humans during passive picture viewing. By making use of various physiological measures, Lang et al. (1997) was one of the first to show in humans that passive-viewing paradigms of aversive pictures can mimic animals’ post-encounter stage of threat, where freezing is the most prominent defensive response (Fanselow, 1994; Bradley et al., 2001). Later, actual posturographic analyses, using a stabilometric force-platform, confirmed that viewing of aversive (vs. neutral and appetitive) stimuli could induce significant reductions in body sway as well as heart rate and reduction in body-sway has been introduced as a proxy to operationalize freezing-like behavior in humans (Azevedo et al., 2005; Facchinetti et al., 2006; Stins and Beek, 2007; Lopes et al., 2009; Roelofs et al., 2010; Hagenaars et al., 2012). Using these methods, retrospective self-reported experiences of aversive life events were found to be associated with increased freezing-like behavior (Hagenaars et al., 2012). However, it remains unstudied whether insecure infant-parent attachment is associated with increased freezing later in life in humans.

The present prospective longitudinal study was designed to examine this relation between quality of infant-parent attachment in infancy and freezing-like behavior in adolescence. We measured freezing in 14-year-old adolescents who are part of the Nijmegen Longitudinal Study and for whom the security of infant-parent attachment had been assessed when they were 15 months old (see Van Bakel and Riksen-Walraven, 2002b). Based on animal work, we predicted that adolescents classified as insecurely attached at 15 months of age, compared to adolescents previously classified as securely attached, would show increased freezing-like behavior in response to social threat, operationalized by angry vs. neutral faces (Roelofs et al., 2010). Angry faces are widely considered to symbolize social threat (Dimberg and Öhman, 1996; Öhman et al., 2001) and have been shown to elicit behavioral, psychophysiological, and neural responses characteristic of a threatening situation, such as increased freezing (Roelofs et al., 2010), startle responses (Anokhin and Golosheykin, 2010), pupil dilation (Kret et al., 2013), skin conductance (Clark et al., 1992), and amygdala reactivity (Whalen et al., 2001; Hermans et al., 2008). To assess whether there is a unique relation for *early* infant-parent attachment, we additionally controlled for the effects of *later* adverse events, including stressful life events and poor quality of parental behavior. Finally, we explored whether the observed freezing-like behavior in adolescents is related to state anxiety as was previously found in adults (Roelofs et al., 2010).

## Materials and Methods

### Participants

The participants of the current study were recruited as part of the Nijmegen Longitudinal Study. This longitudinal study started in 1998 with a community-based sample of 129 children at 15 months of age (Van Bakel and Riksen-Walraven, 2002a) and was regularly followed since then. The families were recruited via local health-care centers in Nijmegen (Netherlands) and were representative of the Dutch population of families with young children (see Van Bakel and Riksen-Walraven, 2002a, b for details of recruitment and family characteristics).

The current study was part of a recent assessment wave at age 14, approved by the local ethics committee (officially called Committee on Research Involving Human Subjects of region Arnhem-Nijmegen). All participants from the previous wave (*n* = 109; age 13) were invited for the 14-year data-collection wave, except for one child diagnosed with multiple psychiatric disorders. Of the 108 participants who received an invitation for the current study, 83 participants (51% boys; *M*_age_ = 14.64 years, *SD* = 0.18) came to the lab to take part in the procedure to measure freezing-like behavior. Out of the 25 participants not participating in this freezing procedure, five could not be contacted and 20 were unwilling or unable to invest time. The freezing assessment was part of a larger, approximately 2.5 h lasting protocol that also included an unrelated fMRI session for 53 of the participants included in this paper, as well as filling in a battery of questionnaires and other perceptual tasks, including pictures of animals, on the stabilometric force-platform (see Figure S1 in Supplementary Material). Prior to testing, participants provided informed assent and parents provided informed consent in accordance with the Declaration of Helsinki. Participants received financial reimbursement for participation. All participants had normal or corrected-to-normal vision. Four participants had to be excluded from the analysis due to technical problems (*n* = 2), dizziness (*n* = 1), or excessive movement (*n* = 1; *z*-score >4 on the posturographic measure). The final sample consisted of 79 participants (49% boys; *M*_age_= 14.63 years, *SD* = 0.18).

Finally, dropout analyses of this longitudinal cohort indicated a slight (though non-significant) tendency of increased dropout rates for participants who were classified as insecurely attached during infancy (see Appendix S1 in online Supplementary Material for statistical details). Even if this effect would have been statistically different, we would not expect any influence of differential dropout on our freezing assessment at age 14.

### Procedures and Measures

#### Infant-Parent Attachment (15 Months of Age)

In the 15-month wave, an abbreviated version of the Strange Situation Procedure (Ainsworth et al., 1978) was used to assess the quality of infant-parent attachment. For a detailed description of the assessment procedure see Van Bakel and Riksen-Walraven (2002b). A similar abbreviated version has been proven valid for the assessment of infant-parent attachment security within normal (Waters et al., 1979; Lewis et al., 1984) as well as within clinical samples (Willemsen-Swinkels et al., 2000). The infants were classified as Secure (B), Avoidant (A), Resistant (C), or Disorganized and Disoriented (D) according to the directions provided by Ainsworth et al. (1978) and Main and Solomon (1990). The inter-rater reliability established on the basis of a randomly selected subset of 20 infants was 95% (see Van Bakel and Riksen-Walraven, 2002b). Of the 79 participants included in the final sample of the present study, 56 participants were classified as Secure (43% boys) and 23 as Insecure (eight Avoidant; seven Resistant; eight Disorganized; 65% boys).

#### Freezing Assessment (Age 14)

A valid and reliable way to assess freezing-like behavior is by means of a stabilometric force-platform, which quantifies spontaneous fluctuations in body sway during threat exposure (Azevedo et al., 2005; Facchinetti et al., 2006; Stins and Beek, 2007; Lopes et al., 2009; Roelofs et al., 2010; Hagenaars et al., 2012). To assess adolescents’ freezing-like behavior, we used a custom-made stabilometric force-platform (dimensions: 50 cm × 50 cm; sampling frequency: 200 Hz; 1 mm accuracy) that measured spontaneous body sway during passive picture viewing. The platform had four force sensors to derive a time series of deviations from center-of-pressure (COP) in the anterior-posterior (AP) and mediolateral (ML) direction. Simultaneously, electrocardiographic (ECG) recordings were collected, using BrainAmp.

Prior to testing, three heart rate electrodes were attached to the skin around the heart of the participants. Next, participants sat in front of a computer screen to watch a short neutral film scene (approximately 3 min), intended to make them feel at ease and to normalize their heart rate. Thereafter, participants were instructed to stand upright and stationary in the middle of the platform, barefooted. We asked them to equally distribute their body weight on both legs with their arms hanging relaxed besides their body. Their feet were next to each other, approximately 20 cm apart. We instructed them to passively look at the pictures presented on the screen and motivated them to keep their attention on the pictures by informing them that remembering the pictures would be important for following tasks. Before the actual task, participants completed a brief practice version in which letters instead of faces were presented on the screen. At the end of the task, a random selection of 18 participants scored the pleasantness of each face stimulus on a seven-point Likert Scale (ranging from 1 = *very angry* to 7 = *very happy*) to check whether the emotional expressions were indeed experienced as angry, neutral, or happy. Both the experimenters and the participants were unaware of the attachment classifications.

This passive-viewing paradigm is based on Roelofs et al. (2010) and consisted of facial emotional expressions: happy, angry, and neutral faces from 20 models (11 male and 9 female) of the Karolinska Directed Emotional Faces database (Lundqvist et al., 1988). Each model expressed each of these facial emotional expressions, resulting in a total of 60 images (20 images per face category). The pictures were presented in gray scale and displayed against a black background. Faces were edited to exclude hair and non-facial features. Each face was presented for 3 s on a 46 × 30 cm^2^ height-adjustable computer screen, approximately 1 m in front of participants, while they were standing on the platform. The face stimuli were presented in three blocks, each consisting of 20 stimuli from the same emotional category, which were presented consecutively without an inter-trial interval. Between blocks, we used an interval of 7 s (5 s black screen followed by 2 s white fixation cross). Block order and stimuli order within blocks were randomized across participants.

#### Intermediate Assessments of Adverse Events

To assess whether there is a unique relation between infant-parent attachment security and later freezing-like behavior, we controlled for the effects of *later* adverse events, including (poor) quality of parental behavior and stressful life events.

The quality of parental behavior was assessed at four intermediate time points, namely at 2$\frac{1}{2}$, 5, 7, and 12 years of age. For each time point trained observers rated standardized videotapes of age-appropriate parent-child interactions on five seven-point scales for the quality of parental interactive behavior (Erickson et al., 1985), namely: (1) supportive presence or the provision of emotional support; (2) respect for the child’s autonomy or non-intrusiveness; (3) effective structure and limit setting; (4) quality of instructions; and (5) hostility. The quality of parental behavior at 2$\frac{1}{2}$ and 5 years has been previously reported (e.g., Smeekens et al., 2007a,b). For the purpose of the present study, we mirrored the hostility scores, standardized each scale per age, and summed these scales to obtain one score for the quality of parental interactive behavior towards the child per age. Higher scores indicate higher quality of parental interactive behavior. When calculating an average overall score of quality of parental behavior across age, missing data (4% missing at age 2$\frac{1}{2}$, 5% missing at age 5, 0% missing at age 7, and 5% missing at age 12) were handled by computing an adjusted score for participants with missing data such that only the non-missing observations were used to compute the score.

The experience of intermediate stressful life events was assessed at 2$\frac{1}{2}$, 5, 7, 9, 12, and 14 years of age. Items from Saranson et al.’s (1978) Life Experiences Survey and Coddington’s (1972) Life Events Scale for Children were selected that were likely to have an aversive influence on the child’s development (e.g., divorce, death of a loved one, serious illness from child or parent). Both scales have been widely used and have sound psychometric properties (Johnston, 1996; Abela, 2001). Parents were asked to respond with “yes” or “no” to all items. These stressful life events have been previously used within this longitudinal study (Smeekens et al., 2007b). For subsequent analyses, we determined an average overall score of experienced life events across all intermediate time points. Missing data (4% missing at age 2$\frac{1}{2}$; 3% missing at age 5; 25% missing at age 7; 9% missing at age 9; 3% missing at age 12; and 1% missing at age 14) were handled in the same way as the quality of parental behavior.

#### State Anxiety

State anxiety, was assessed before the freezing assessment using a self-report visual analog (VAS) scale where participants indicated on a scale from 0–100 (0 = *not anxious at all*; 100 = *extremely anxious*) how anxious they felt at that moment. Data were missing for four participants; for three participants we relied on a previous assessment filled in approximately 1 h before.

### Data Analysis

#### Posturography

Posturographic analyses were performed with MATLAB (MathWorks, Natick, MA, USA). First, we calculated for each participant the mean position of the COP in the AP and ML direction per time point. Based on this, we computed each participant’s variability in body sway—separately for each emotional block—expressed as the standard deviation of the COP in the AP (SD-AP) and the ML (SD-ML) direction (Roelofs et al., 2010). The resulting variability in body sway (SD-AP, SD-ML) represents an average of participants’ body movement during the 1-min presentation period of facial emotional expressions from the same emotional category. Lower scores indicate *increased* postural immobility. Importantly, participants’ relatively stable body posture on the stabilometric force-platform leads to a larger range of movements in the AP than the ML direction (Roelofs et al., 2010). This makes the AP fluctuations more sensitive to affective processes compared to the ML fluctuations. Consistent with our expectation based on previous literature (Roelofs et al., 2010) we briefly confirmed that body sway of SD-ML was indeed not modulated by Emotion (Greenhouse-Geisser correction: *F*_(1.85,143.87)_ = 1.59, *p* = 0.208, ${\text{η}}_{p}^{2}$ = 0.02). Accordingly, we used SD-AP to operationalize body sway and to assess the influence of attachment on body sway.

#### Heart Rate

By using brainvision (Analyzer 2.0), mean heart rate in beats per minute (BPM) was determined separately for the three emotional blocks.

#### Statistical Analyses

First, a separate Friedman’s Test was conducted to confirm that the presented emotional faces were indeed rated as angry, happy, and neutral, respectively. Second, we used a log transformation to reduce skewness of the body-sway measure (SD-AP). Heart rate (BPM) and log transformed body sway were then analyzed with separate repeated-measures ANOVAs, with Emotion (happy, angry, neutral) as within-subject factor and Attachment security (secure, insecure) as between-subject factor. Importantly, previous work has shown that freezing-like behavior in response to angry faces has to be evaluated relative to neutral or happy faces in order to control for individual differences in body sway and heart rate (see Azevedo et al., 2005; Facchinetti et al., 2006; Stins and Beek, 2007; Lopes et al., 2009; Roelofs et al., 2010; Hagenaars et al., 2012). Accordingly, the crucial test of our prediction is a significant Emotion × Attachment interaction. Significant main and interaction effects were further investigated in follow-up analyses. Pearson correlations were further used to assess the previously found relation between threat-related reductions in body sway and heart rate (Roelofs et al., 2010). To assess whether the relation between attachment security and freezing-like behavior is unique for *early* infant-parent attachment, we controlled for later adverse events by adding Quality of Parental Behavior and the experience of Stressful Life Events as separate covariates into the model. Finally, we tested whether State Anxiety was related to adolescents’ freezing-like behavior to replicate previous work in adults by Roelofs et al. (2010). Because of unequal sizes of attachment groups, we tested for each ANOVA whether the error variance and the observed covariance matrices of the dependent variable (SD-AP, heart rate) were equal across both groups. Levene’s Test of Equality of Error Variances and Box’s Test of Equality of Covariance Matrices of all analyses were non-significant (all *p*s > 0.16), suggesting an equal error variance and equal covariance matrices of SD-AP and heart rate across attachment groups.

## Results

### Manipulation Check

A Friedman test, evaluating differences in pleasantness ratings on a seven-point Likert Scale (collected in a random selection of 18 participants) for angry, happy, and neutral faces, revealed a significant effect of Emotion (χ^2^_(2,*N* = 18)_ = 36, *p* < 0.001). Follow-up pairwise (Bonferroni corrected) comparisons indicated that as expected each emotional category differed significantly from the other two emotional expressions with happy > neutral > angry (all *p*s < 0.01; happy: *M* = 5.94, *SD* = 0.22; neutral: *M* = 3.95, *SD* = 0.17; angry: *M* = 2.25, *SD* = 0.35).

### Body Sway

The ANOVA with body-sway (SD-AP) as dependent variable, Emotion (angry, happy, neutral) as within-subject factor, and Attachment (secure, insecure) as between-subject factor showed a significant main effect of Emotion (*F*_(2,76)_ = 4.74, *p* = 0.011, ${\text{η}}_{p}^{2}$ = 0.11) but not for Attachment (*F*_(1,77)_ = 0.92, *p* = 0.341, ${\text{η}}_{p}^{2}$ = 0.01). Most importantly, the Emotion × Attachment interaction was significant (*F*_(2,76)_ = 3.23, *p* = 0.045, ${\text{η}}_{p}^{2}$ = 0.08; see Table 1 for the estimated means, separately for attachment security and emotional valence).

**Table 1:** **Posturographic data of anterior-posterior sway (SD-AP in mm)^a^ as a function of Emotion (Neutral/Happy/Angry) and Attachment security (Insecure/Secure)**.

	Attachment security			
	Insecure (*n* = 23)		Secure (*n* = 56)	
Faces	*M (SEM)*	95% CI	*M (SEM)*	95% CI
Neutral	0.44 (0.03)	[0.37, 0.51]	0.38 (0.02)	[0.33, 0.42]
Happy	0.40 (0.03)	[0.34, 0.46]	0.36 (0.02)	[0.33, 0.40]
Angry	0.37 (0.03)	[0.30, 0.43]	0.37 (0.02)	[0.33, 0.41]

*Notes: *M* = marginal mean; CI = confidence interval; resulting from ANOVA with body-sway (SD-AP) as dependent variable, Emotion (angry, happy, neutral) as within-subject factor, and Attachment (secure, insecure) as between-subject factor. ^a^log transformed*.

**Figure 1:**
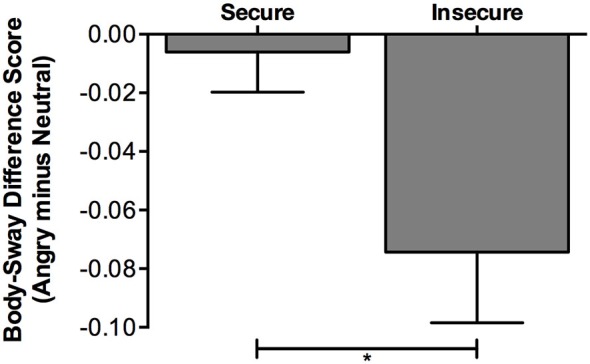
**Differences in log-transformed body-sway variability while viewing angry compared to neutral faces, separately for insecurely and securely attached participants**. Body sway is expressed in standard deviation from participants’ center-of-pressure (COP) in the anterior-posterior direction (SD-AP). Larger negative difference scores (SD-AP for angry faces minus SD-AP for neutral faces) reflect stronger freezing-like reactions in response to angry compared to neutral faces. Error bars represent standard errors. *p < 0.05; Secure: 95% CI [−0.03, 0.02]; Insecure: 95% CI [−0.12, −0.03].

We explored the critical Emotion × Attachment interaction by assessing which of the three Emotion contrasts interacted significantly with Attachment. In line with our expectations, the *angry vs. neutral* Emotion contrast showed a significant interaction with Attachment (*F*_(1,77)_ = 6.52, *p* = 0.013, ${\text{η}}_{p}^{2}$ = 0.08; Secure: *M*_difference_ = −0.01, *SEM* = 0.01, 95% CI [−0.03, 0.02]; Insecure: *M*_difference_ = −0.07, *SEM* = 0.02, 95% CI [−0.12, −0.03]); results are illustrated in Figure 1), whereas we did not find significant interaction effects with attachment for the two other Emotion contrasts: (i) *angry vs. happy*: *F*_(1,77)_ = 3.02, *p* = 0.086, ${\text{η}}_{p}^{2}$ = 0.04 (Secure: *M*_difference_ = 0.01, *SEM* = 0.01, 95% CI [−0.02, 0.03]; Insecure: *M*_difference_ = −0.04, *SEM* = 0.02, 95% CI [−0.08, 0.00]); (ii) *happy vs. neutral*: *F*_(1,77)_ = 1.43, *p* = 0.235, ${\text{η}}_{p}^{2}$ = 0.02 (Secure: *M*_difference_ = −0.01, *SEM* = 0.01, 95% CI [−0.03, 0.01]; Insecure: *M*_difference_ = −0.04, *SEM* = 0.02, 95% CI [−0.07, 0.00]).

Next, we explored the Emotion × Attachment effect for the critical *angry-neutral* Emotion contrast, by testing this Emotion contrast in each Attachment group separately. As expected, these analyses revealed a significant effect of Emotion in the insecurely attached participants but not in the securely attached participants: (i) insecurely attached: *F*_(1,22)_ = 9.16, *p* = 0.006, ${\text{η}}_{p}^{2}$ = 0.29; angry: *M* = 0.37, *SEM* = 0.03, 95% CI [0.30, 0.44]; neutral: *M* = 0.44, *SEM* = 0.04, 95% CI [0.36, 0.52]; (ii) securely attached: *F*_(1,55)_ = 0.20, *p* = 0.661, ${\text{η}}_{p}^{2}$ = 0.00; angry: *M* = 0.37, *SEM* = 0.02, 95% CI [0.33, 0.41]; neutral: *M* = 0.38, *SEM* = 0.02, 95% CI [0.34, 0.41].

*Post hoc* comparisons (Bonferroni corrected) for the main effect of Emotion (angry: *M* = 0.37, *SEM* = 0.02, 95% CI [0.33, 0.41]; happy: *M* = 0.38, *SEM* = 0.02, 95% CI [0.35, 0.42]; neutral: *M* = 0.41, *SEM* = 0.02, 95% CI [0.37, 0.45]) revealed a significant effect for the *angry vs. neutral* contrast (*p* = 0.011), but not for the *happy vs. neutral* contrast (*p* = 0.094) and the *angry vs. happy* contrast (*p* = 0.549).

For the sake of completeness, we tested whether the insecurely attached adolescents differed from their securely attached counterparts in body sway for each of the facial emotional expressions separately. There was no significant effect of Attachment on SD-AP, for angry (*F*_(1,77)_ = 0.01, *p* = 0.944, ${\text{η}}_{p}^{2}$ = 0.00), neutral (*F*_(1,77)_ = 2.65, *p* = 0.108, ${\text{η}}_{p}^{2}$ = 0.03), or happy faces (*F*_(1,77)_ = 1.19, *p* = 0.278, ${\text{η}}_{p}^{2}$ = 0.02). This suggests that Attachment security does not have a general effect on body sway and confirms that effects on freezing-like behavior need to be evaluated in terms of relative differences in SD-AP between emotional categories.

As secure vs. insecure attachment groups differed in gender distributions, we conducted the same analyses including Gender separately as a covariate and as a between-subject factor. The critical results remained significant when controlling for Gender (see Appendix S2 in online Supplementary Material).

### Heart Rate

**Figure 2:**
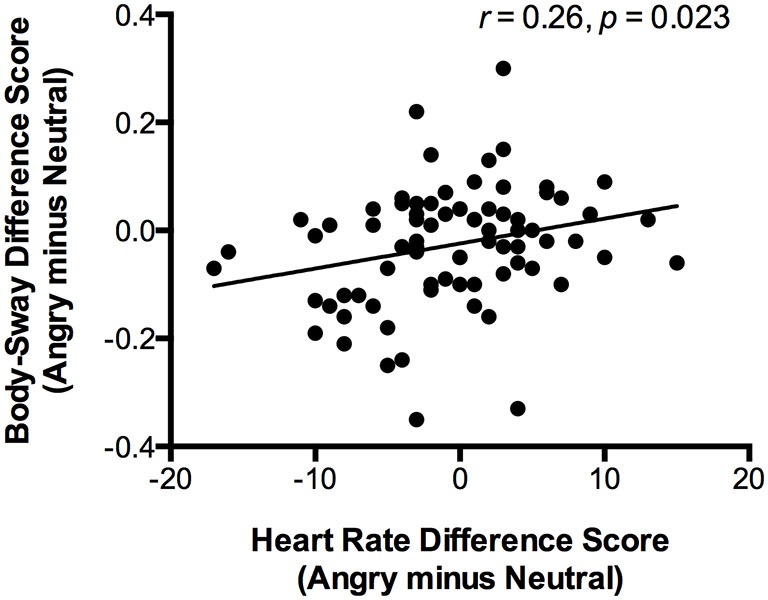
**The scatter plot (with best-fitting regression line) illustrates the correlation between change in heart rate variability (in beats per minute) and change in body-sway variability (in mm) while participants viewed angry compared to neutral faces**. Change in heart rate variability was calculated by subtracting heart rate of the neutral-faces block from the same measure of the angry-faces block. The same calculation was applied to conduct body-sway variability.

The sample for heart rate data contained one participant less than the body-sway data due to technical problems in assessing heart rate for this one participant. An ANOVA with heart rate (BPM) as dependent variable, Emotion (angry, happy, neutral) as within-subject factor, and Attachment (secure, insecure) as between-subject factor revealed no main or interaction effects (Emotion: *F*_(2,75)_ = 0.45, *p* = 0.638, ${\text{η}}_{p}^{2}$ = 0.01; Emotion × Attachment interaction: *F*_(2,75)_ = 0.75, *p* = 0.478, ${\text{η}}_{p}^{2}$ = 0.02; Attachment: *F*_(1,76)_ = 2.08, *p* = 0.153, ${\text{η}}_{p}^{2}$ = 0.03), suggesting that the heart rate reduction for angry vs. neutral faces was not significant. However, the body-sway reduction for angry vs. neutral faces was significantly correlated to a heart rate reduction for angry vs. neutral faces (*r* = 0.26, *p* = 0.023), indicating that the threat-related reduction in body sway was accompanied by a threat-related reduction in heart rate (Figure 2).

### Later Adverse Events

To determine whether there is a unique relation for *early* infant-parent attachment, we controlled for the effects of *later* adverse events, including Stressful Life Events and Quality of Parental Behavior. The critical results remained significant when including these variables as covariates in the model: the Emotion × Attachment interaction for the three emotions remained marginally significant (*F*_(2,74)_ = 3.02, *p* = 0.055, ${\text{η}}_{p}^{2}$ = 0.08), which was, as before, specific for the *angry vs. neutral* Emotion contrast (*F*_(1,75)_ = 6.11, *p* = 0.016, ${\text{η}}_{p}^{2}$ = 0.08) as well as for the insecurely attached participants (*F*_(1,20)_ = 6.48, *p* = 0.019, ${\text{η}}_{p}^{2}$ = 0.25; see Appendix S3 in online Supplementary Material for detailed descriptions of results). This suggests a unique relation between *early* insecure infant-parent attachment and *later* freezing-like behavior that cannot be explained by later-occurring life events or quality of parental behavior.

### State Anxiety

**Figure 3:**
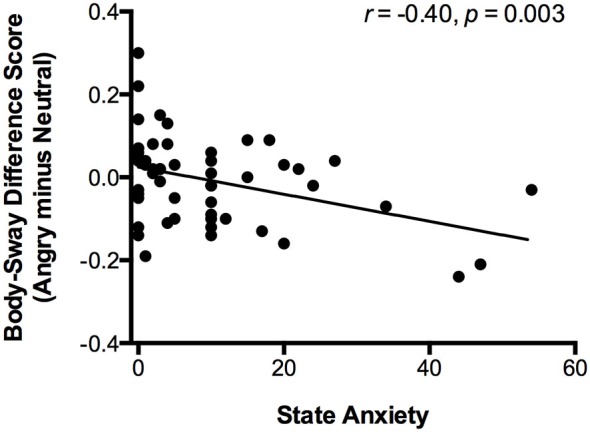
**The scatter plot (with best-fitting regression line) shows the correlation between state anxiety and change in body-sway variability (in mm) of angry vs. neutral faces**. Change in body-sway variability was calculated by subtracting log-transformed body sway of the neutral-faces block from log-transformed body sway of the angry-faces block.

To replicate previous work by Roelofs et al. (2010), we repeated the ANOVA with SD-AP as dependent variable, Emotion (angry, happy, neutral) as within-subject factor, Attachment (secure, insecure) as between-subject factor and included this time State Anxiety as a covariate. Similar as before, we found a marginally significant Emotion × Attachment interaction (*F*_(2,74)_ = 2.87, *p* = 0.063, ${\text{η}}_{p}^{2}$ = 0.07; Emotion: *F*_(2,74)_ = 1.29, *p* = 0.281, ${\text{η}}_{p}^{2}$ = 0.03; Attachment: *F*_(1,75)_ = 0.90, *p* = 0.345, ${\text{η}}_{p}^{2}$ = 0.01), which was specific for the *angry vs. neutral* comparison (*F*_(1,75)_ = 5.78, *p* = 0.019, ${\text{η}}_{p}^{2}$ = 0.07; angry vs. happy: *F*_(1,75)_ = 2.86, *p* = 0.095, ${\text{η}}_{p}^{2}$ = 0.04; happy vs. neutral: *F*_(1,75)_ = 1.10, *p* = 0.297, ${\text{η}}_{p}^{2}$ = 0.01) as well as for the previously insecurely attached participants (insecure: *F*_(1,21)_ = 12.68, *p* = 0.002, ${\text{η}}_{p}^{2}$ = 0.38; secure: *F*_(1,53)_ = 2.35, *p* = 0.132, ${\text{η}}_{p}^{2}$ = 0.04). All analyses revealed no main effect of State Anxiety (all *p*s > 0.40) as well as no State Anxiety × Emotion interaction (all *p*s > 0.08), except for the follow-up analyses separately for the securely attached participants (*F*_(1,53)_ = 9.82, *p* = 0.003, ${\text{η}}_{p}^{2}$ = 0.16). To follow-up on this State Anxiety × Emotion interaction in the securely attached group, we calculated the correlation between the body-sway difference score of angry vs. neutral faces and State Anxiety. A negative correlation (*r* = −0.40, *p* = 0.003) indicated that higher State Anxiety in securely attached participants was associated with greater body-sway reduction for angry vs. neutral faces (see Figure 3). Thus, likewise previous observations by Roelofs et al. (2010) in an unselected adult sample, we found a correlation between State Anxiety and body-sway reduction for angry relative to neutral faces.

## Discussion

The results of this prospective longitudinal study show that insecure infant-parent attachment, assessed at 15 months of age, is associated with increased freezing-like behavior in adolescence. Adolescents who were classified as insecurely attached in infancy, compared to those who were securely attached, showed significantly stronger freezing-like behavior as indicated by reduced body sway in response to angry relative to neutral faces. Notably, in the securely attached group, freezing-like behavior was only observed in those individuals scoring high on state anxiety. Furthermore, later quality of parental behavior and later experience of stressful life events could not explain the relation between early insecure attachment and increased freezing-like behavior during adolescence. These results suggest a specific role of *early* infant-parent attachment security in shaping the human defensive response systems.

The observed body-sway reduction of angry relative to neutral faces in the insecurely attached group—compared to the securely attached group—seems to be driven by the quantity of body sway in response to neutral faces (our baseline condition) rather than less body sway in response to angry faces. This increase in basal body sway may be indicative of increased basal restlesness or impulsivity, which has been observed in insecurely attached children (Olson et al., 1990; Jacobsen et al., 1997). Alternatively, it can be speculated that neutral (vs. angry) facial emotional expressions capture less attention in insecurely (compared to securely) attached individuals for example because those faces might contain little emotionally relevant information of importance for the activation of their attachment system (Bowlby, 1980). Importantly, the relatively higher amount of basal body sway in response to neutral faces observed in the insecurely attached group—regardless of whether it reflects restlessness or decreased attention capture—is not present when viewing *angry* faces. This finding is compatible with the current notion of freezing, where freezing is regarded as a relative reduction of body sway in response to aversive relative to neutral information (Azevedo et al., 2005; Facchinetti et al., 2006; Stins and Beek, 2007; Roelofs et al., 2010; Hagenaars et al., 2012).

This interpretation is consistent with the notion that episodes of freezing in threat extinction in rats and other rodents are embedded within gradually increasing instances of bodily activity (Curti, 1935; Bolles and Collier, 1976). Our results are also consistent with research in animals that suggests long-lasting effects of early stressful life experiences in rat pups’ sensitive attachment learning period (Sullivan and Holman, 2010) on primary defensive stress-responses, including increased freezing (Menard et al., 2004; Sanders and Knoepfler, 2008). Particularly, life-events related to poor parenting behavior during this sensitive period have been associated with increased freezing to later threatening encounters in rodents (Caldji et al., 2000; Menard et al., 2004). The results also support predictions of a human early life stress model (Loman and Gunnar, 2010), which proposes that insecure infant-parent attachment can result in increased defensive stress-responses, including increased freezing in the face of threatening experiences.

For the securely attached individuals, we found no evidence for freezing-like behavior in our primary analysis. However, when including state anxiety in the model, it became clear that in this particular group, the reduced body-sway for angry vs. neutral faces was only present in the high anxious and not in the low anxious individuals. The finding of a significant correlation between state anxiety and freezing-like behavior is consistent with previous results on freezing in *adults* (Roelofs et al., 2010). These authors showed a similar relation between state anxiety and body-sway reduction of angry vs. neutral faces in the same measure that we used here (standard deviation of the center-of-pressure in the anterior-posterior direction, SD-AP) and elsewhere (e.g., Lopes et al., 2009; Hagenaars et al., 2012). Replicating these results provides initial indications that defensive stress-responses can be equally well objectively assessed and quantified in developmental populations with lower body weight as in adults. Our observed Emotion effect for the angry vs. neutral contrast in the whole sample is comparable in terms of absolute body-sway difference (0.18 mm, untransformed) to previous work [0.14 mm reported by Roelofs et al. (2010)]. Additionally, the effect size of our Attachment × Emotion effect for the angry vs. neutral contrast (${\text{η}}_{p}^{2}$ = 0.08) is comparable, but somewhat smaller, to the effect size of a similar Emotion × Group interaction [${\text{η}}_{p}^{2}$ = 0.16 reported by Hagenaars et al. (2012)].

The result that adolescents who were classified as being insecurely attached in infancy show increased threat-related freezing to angry vs. neutral faces is particularly interesting in light of research suggesting that insecure infant-parent attachment is a risk factor for developmental problems and psychopathologies later in life (Renken et al., 1989; Warren et al., 1997; Burgess et al., 2003; Shamir-Essakow et al., 2005; Smeekens et al., 2007b; Colonnesi et al., 2011; Groh et al., 2012). On the basis of a number of observations, we propose that increased freezing may constitute an intermediate risk factor for psychopathology (for a review, see also Hagenaars et al., 2014): studies in animals and humans have indicated that freezing serves to optimize the perceptual system for detecting cues when threat is still at relative distance by enhancing attention and reducing body sway (Blanchard et al., 1986; Lang et al., 1997). This vigilance-enhancing state—thought to optimize coping strategies in healthy subjects—may get out of balance after chronic stress due to insecure infant-parent attachment (Loman and Gunnar, 2010). Enhanced vigilance for threat and fear-evoking cues may in turn enhance encoding of traumatic events (Ehlers and Clark, 2000; Naim et al., 2013). Such mechanisms may explain the previously found relation between peritraumatic freezing behavior and post-traumatic stress symptoms reported by trauma victims (Bovin et al., 2008). However, this hypothesis is still speculative and future research, including attentional and memory tests, is needed to test it. Also it should be mentioned that although increased freezing tendencies may be a risk factor in some children, it might as well serve as an adaptive coping strategy and resilience factor in other children (Hagenaars et al., 2014). It could for example represent an adaptation or sensitization to the environment of chronic stress (Frankenhuis and de Weerth, 2013). Future research should investigate whether freezing may moderate or mediate the previously found relation between insecure infant-parent attachment on the one hand and the development of maladaptive stress coping and psychopathology on the other hand.

A few caveats should be considered when interpreting the current results. First, it is important to note that there are substantial individual differences in the general amount of body sway (Yamamoto et al., 2015). Therefore, absolute body sway in response to angry faces cannot be interpreted independently as freezing-like behavior, but must be interpreted relative to an emotional neutral category (such as neutral faces) to control for individual differences in the amount of body sway. Contrasting the within-subject conditions as we did is the standard way to analyze such data in order to obtain a sensitive measure of bodily freezing-like behavior (Azevedo et al., 2005; Facchinetti et al., 2006; Stins and Beek, 2007; Lopes et al., 2009; Roelofs et al., 2010; Hagenaars et al., 2012). Nevertheless, future studies could benefit from including a second lower-level baseline (e.g., a non-social neutral condition) randomly displayed throughout the task or a trial-based baseline measure before the presentation of each face to more directly investigate the individual differences in baseline body-sway.

Second, although the magnitude of the body-sway reduction for angry vs. neutral faces was significantly correlated to the heart rate reduction for angry vs. neutral faces, there were no relations between attachment and heart rate changes.

Third, future research should investigate which moderating (e.g., genetic) factors and mediating (e.g., epigenetic) factors (Claessens et al., 2011) as well as which cognitive processes, like (sub)conscious appraisal (Öhman et al., 2000; Sander et al., 2007), attention, and memory processes, may contribute to the explanation of the observed relation between attachment and freezing. Accordingly, future longitudinal investigations may profit from collecting genetic or epigenetic data when studying the relation between quality of infant-parent attachment and primary defensive stress-reactions.

Fourth, only a subset of participants rated the pleasantness of each of the facial emotional expressions. In future research it would be interesting to investigate whether freezing differences between attachment groups might be explained by differences in the perception and evaluation of neutral or emotional faces. Additionally, future research should investigate whether the experienced dominance of the facial expressions, particularly regarding angry faces, plays a role in the observed relation between attachment and freezing.

Fifth, freezing in animals provides an important theoretical framework for human freezing and has been shown to have substantial overlap with human freezing-like behavior (reviewed by Hagenaars et al., 2014), such as being expressed by similar bodily features (Fanselow, 1984; Roelofs et al., 2010), being elicited in similar situations (for review, see Hagenaars et al., 2014), showing similar neural correlates (Gozzi et al., 2010; Hermans et al., 2013), and being amplified by traumatic events and anxiety in both animals and humans (Bovin et al., 2008; Sanders and Knoepfler, 2008; Roelofs et al., 2010; Hagenaars et al., 2012). Nevertheless, it is important to remain cautious when comparing freezing of animals to human freezing-like behavior because of different assessment procedures and potential differences in underlying mechanisms and functions. More translational work is needed to fully understand how animal and human freezing are related.

Finally, the group size of adolescents classified as insecurely attached infants did not allow analyses of the different *subtypes* of insecure attachment (Avoidant, Resistant, Disorganized), which may be associated with different types of non-optimal parental caregiving (e.g., Carlson et al., 2003). Therefore, it would be interesting to investigate in future studies with larger sample sizes whether different types of insecure attachment have different implications for freezing.

In conclusion, the results of this prospective longitudinal study suggest that insecure infant-parent attachment is associated with adolescents’ increased freezing-like behavior to angry relative to neutral faces. This relation could not be explained by the intermediate quality of parental interactive behavior as well as the intermediate experience of stressful life events, supporting the notion of infancy as a sensitive time window in which early parental responsiveness and sensitivity to the infants’ needs are essential in shaping the human defensive stress-response system. Additionally, our results extend existing knowledge by providing the first empirical indications suggesting that early infant-parent attachment is related to primary defensive reactions not only in animals but also in humans. Furthermore, it is also noteworthy that the attachment-related differences in freezing-like behavior can be objectively assessed during early adolescence, 13 years after the infants had been classified as securely or insecurely attached. Given the previously observed relation between freezing during trauma and the development of post-traumatic stress symptoms in adults (Bovin et al., 2008) and given the consideration of freezing as a trait variable in research with animals (Qi et al., 2010), it is important for future research to replicate current results and to start investigating the role of freezing as an intermediate factor in the development of psychopathology. The currently presented experimental tool opens venues to start exploring this relation in an ecologically valid and reliable way in humans.

## Author Contributions

All authors contributed to the study concept and design. The Nijmegen Longitudinal Study was initiated by MR, who also supervised the 15-month assessment wave. SS supervised the data collection at age 14. Freeze assessment was performed by HN. HN and VL performed data analysis and interpretation under the supervision of KR, with input by BF. HN drafted the paper and all others provided critical revisions. All authors approved the final version of the paper prior to submission.

## Funding

HN was supported by a Research Assistant Grant [705AO-2379] from the Royal Netherlands Academy of Arts and Sciences and by a Research Talent Grant [406-13-022] from the Netherlands Organization for Scientific Research (NWO). KR was funded by a VICI grant [#453-12-001] from NWO and a starting grant from the European Research Council [ERC_StG2012_313749]. The content is solely the responsibility of the authors and does not necessarily represent the official views of the funding agencies.

## Conflict of Interest Statement

The authors declare that the research was conducted in the absence of any commercial or financial relationships that could be construed as a potential conflict of interest.
