# Supplementary material for: Infant attachment predicts bodily freezing in adolescence: evidence from a prospective longitudinal study
Source: Front Behav Neurosci. 2015 Oct 12;9:263. doi: 10.3389/fnbeh.2015.00263 (PMC4617177; doi:10.3389/fnbeh.2015.00263)
Supplement: Supplementary file 1 [file Datasheet_1.doc]

***Supplementary Material***

**Infant attachment predicts bodily freezing in adolescence: evidence from a prospective longitudinal study**

**Hannah C.M. Niermann^*^, Verena Ly, Sanny Smeekens, Bernd Figner, J. Marianne Riksen-Walraven, Karin Roelofs**

*** Correspondence:** Hannah C.M. Niermann: h.niermann@psych.ru.nl

**Appendix S1 - Dropout analyses**

To determine whether the originally recruited longitudinal sample at 15-months of age differed in attachment security from the current analytic sample at age 14, we conducted a Pearson Chi-Square test, indicating a slight (although non-significant) tendency of increased dropout rates for participants who were classified as insecurely attached during infancy (χ^2^_(1_,*_N_*_= 127)_ = 3.65, *p* = 0.056; percentage attachment security of originally longitudinal participants lost at age 14: 54% secure, 46% insecure; percentage attachment security of current analytic sample: 71% secure, 29% insecure).

**Appendix S2 - Body Sway, Attachment, and Gender**

To investigate whether the reported body-sway results can be explained by the uneven gender distribution among the secure vs. insecure attachment groups, we repeated the previous ANOVA with SD-AP as dependent variable, Emotion (angry, happy, neutral) as within-subject factor, Attachment (secure, insecure) as between-subject factor, but this time we included Gender as a covariate. This analysis revealed a marginal significant Emotion × Attachment interaction (*F*_(2,75)_ = 3.02, *p* = 0.055, *η_p_****^2^*** = 0.07), a marginal significant main effect of Emotion (*F*_(2,75)_ = 2.72, *p* = 0.073, *η_p_****^2^*** = 0.07), and a non-significant main effect of Attachment (*F*_(1,76)_ = 0.18, *p* = 0.671, *η_p_****^2^*** = 0.00). Most importantly, however, and replicating the results of the analyses without Gender reported in the main text, the Emotion × Attachment interaction in the *angry vs. neutral* ANOVA was significant (*F*_(1,76)_ = 6.11, *p* = 0.016, *η_p_****^2^*** = 0.07), but, again as before, not in the other two—*angry vs. happy* and *happy vs. neutral*—ANOVAs (angry vs. happy: *F*_(1,76)_ = 2.68, *p* = 0.106, *η_p_****^2^*** = 0.03; happy vs. neutral: *F*_(1,76)_ = 1.46, *p* = 0.230, *η_p_****^2^*** = 0.02). Further replicating the results in the main text, the interaction for the Emotion contrast of *angry vs. neutral* faces was significant for the insecurely attached participants (*F*_(1,21)_ = 8.98, *p* = 0.007, *η_p_****^2^*** = 0.30), but not for the securely attached participants (*F*_(1,54)_ = 0.02, *p* = 0.879, *η_p_****^2^*** = 0.00). All analyses—except for the follow-up analysis separately for the insecurely attached participants—revealed a significant main effect of gender (all *p*s < 0.04), showing that boys moved on average more compared to girls. However, none of these analyses revealed a significant Emotion × Gender interaction (all *p*s > 0.35). Levene’s Test of Equality of Error Variances and Box’s Test of Equality of Covariance Matrices of all analyses were non-significant (all *p*s > 0.16), suggesting an equal error variance and equal covariance matrices of SD-AP across attachment groups.

We repeated the ANOVA with SD-AP as dependent variable, Emotion (angry, happy, neutral) as within-subject factor, Attachment (secure, insecure) as between-subject factor, but this time included Gender as a between-subject factor. This analysis again showed a marginal significant Emotion x Attachment interaction (*F*_(2,74)_ = 2.48, *p* = 0.091, *η_p_****^2^*** = 0.06; Emotion: *F*_(2,74)_ = 3.37, *p* = 0.040, *η_p_****^2^*** = 0.08; Attachment: *F*_(1,75)_ = 0.16, *p* = 0.690, *η_p_****^2^*** = 0.00), which was, as before, specific for the angry vs. neutral contrast (*F*_(1,75)_ = 4.95, *p* = 0.029, *η_p_****^2^*** = 0.06; angry vs. happy: *F*_(1,75)_ = 2.73, *p* = 0.103, *η_p_****^2^*** = 0.04; happy vs. neutral: *F*_(1,75)_ = 0.78, *p* = 0.379, *η_p_****^2^*** = 0.01) and for the insecurely attached participants (insecure: *F*_(1,21)_ = 6.70, *p* = 0.017, *η_p_****^2^*** = 0.24; secure: *F*_(1,54)_ = 0.12, *p* = 0.727, *η_p_****^2^*** = 0.00). All analyses revealed no Emotion x Gender interaction (all *p*s > 0.35), no Gender x Attachment interaction (all *p*s > 0.76), and no Emotion x Gender x Attachment interaction (all *p*s > 0.07). As before, all analyses—except for the follow-up analysis separately for the insecurely attached participants—showed a main effect of gender (all *p*s < 0.04). Levene’s Test of Equality of Error Variances and Box’s Test of Equality of Covariance Matrices of all analyses were non-significant (all *p*s > 0.05), suggesting an equal error variance and equal covariance matrices of SD-AP across attachment and gender groups. Overall, these analyses revealed similar results compared to the analyses including Gender as a covariate.

In sum, these results show that the uneven gender distribution cannot explain the results reported in the main text.

**Appendix S3 - Body Sway, Attachment, Quality of Parental Behavior, and the Experience of Stressful Life Events**

To test whether there is a unique relation between the reported body-sway results and attachment security, we repeated the analyses reported in the main text with SD-AP as dependent variable, Emotion (angry, happy, neutral) as within-subject factor, Attachment (secure, insecure) as between-subject factor, but this time we included the intermediate Quality of Parental Behavior as well as the intermediate experience of Stressful Life Events as covariates in the model. This ANOVA revealed a marginal significant Emotion x Attachment interaction (*F*_(2,74)_ = 3.02, *p* = 0.055, *η_p_****^2^*** = 0.08; Emotion: *F*_(2,74)_ = 1.32, *p* = 0.274, *η_p_****^2^*** = 0.03; Attachment: *F*_(1,75)_ = 0.44, *p* = 0.509, *η_p_****^2^*** = 0.01), which was, as before, specific for the *angry vs. neutral* comparison (*F*_(1,75)_ = 6.11, *p* = 0.016, *η_p_****^2^*** = 0.08), but not for the *angry vs. happy* and *happy vs. neutral* comparisons (angry vs. happy: *F*_(1,75)_ = 2.74, *p* = 0.102, *η_p_****^2^*** = 0.04; happy vs. neutral: *F*_(1,75)_ = 1.45, *p* = 0.232, *η_p_****^2^*** = 0.02). Furthermore, the interaction for the Emotion contrast of *angry vs. neutral* remained significant for the insecurely attached participants (*F*_(1,20)_ = 6.48, *p* = 0.019, *η_p_****^2^*** = 0.25), but not for their securely attached counterparts (*F*_(1,53)_ = 1.35, *p* = 0.250, *η_p_****^2^*** = 0.03). All analyses revealed no main effect of Quality of Parental Behavior (all *p*s > 0.19) and no Emotion x Quality of Parental Behavior interaction (all *p*s > 0.47). Furthermore, all analyses showed no main effect of Stressful Life Events (all *p*s > 0.06) and no Emotion x Stressful Life Events interaction (all *p*s > 0.06). The critical results remained significant when excluding a participant because of three missing assessment points of Quality of Parental Behavior and four missing assessment points of Stressful Life Events. Levene’s Test of Equality of Error Variances and Box’s Test of Equality of Covariance Matrices of all analyses were non-significant (all *p*s > 0.16), suggesting an equal error variance and equal covariance matrices of SD-AP across attachment groups.

**Excluding stressful life events reported at age 7.** Because of 25% missing scales at age 7, we repeated the above-mentioned analyses, however, this time we excluded the experience of Stressful Life Events reported at age 7 when calculating the overall means for the experience of Stressful Life Events across age. The critical results remained (Attachment x Emotion: *F*_(2,74)_ = 3.08, *p* = 0.052, *η_p_****^2^*** = 0.08; Emotion: *F*_(2,74)_ = 0.81, *p* = 0.447, *η_p_****^2^*** = 0.02; Attachment: *F*_(1,75)_ = 0.41, *p* = 0.523, *η_p_****^2^*** = 0.01): The Attachment x Emotion interaction was specific for the *angry vs. neutral* comparison (*F*_(1,75)_ = 6.24, *p* = 0.015, *η_p_****^2^*** = 0.08; angry vs. happy: *F*_(1,75)_ = 2.73, *p* = 0.103, *η_p_****^2^*** = 0.04; happy vs. neutral: *F*_(1,75)_ = 1.50, *p* = 0.224, *η_p_****^2^*** = 0.02) and specific for the insecurely attached individuals (*F*_(1,20)_ = 6.59, *p* = 0.018, *η_p_****^2^*** = 0.25; securely attached: *F*_(1,53)_ = 1.56, *p* = 0.217, *η_p_****^2^*** = 0.03). All analyses indicated no main effect of Quality of Parental Behavior (*p*s > 0.20) and no Emotion x Quality of Parental Behavior interaction (*p*s > 0.44). Additionally, all analyses showed no main effect of Stressful Life Events (*p*s > 0.06) and no Emotion x Stressful Life Events interaction (*p*s > 0.30; except for the follow-up analysis separately for the securely attached participants, *p* = 0.049). Levene’s Test of Equality of Error Variances and Box’s Test of Equality of Covariance Matrices of all analyses were non-significant (all *p*s > 0.16), suggesting an equal error variance and equal covariance matrices of SD-AP across attachment groups. Overall, the missing scales at age 7 cannot explain the observed results.

**Supplementary Figure S1 – Experimental Procedure**

*
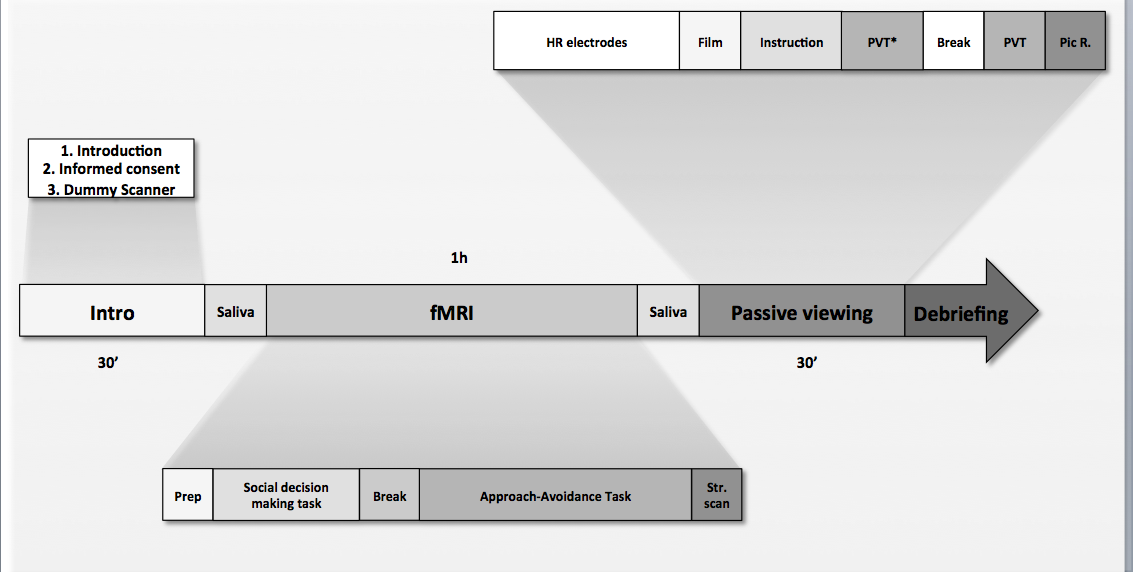
*

**Figure S1 |** **Overview of whole assessment procedure, consisting of a 30-minute introduction phase, a 1 hour fMRI session, including saliva samples and mood assessment before and after fMRI testing, and finally the 30-minute passive viewing/freezing assessment of interest for this paper** (Notes: Prep = Preparation; Str. scan = structural scan; HR = heart rate; PVT = Passive Viewing Task [*first PVT of interest for our study], Pic R. = Picture Rating [for a subset of participants]). The total duration was approximately 2.5 hours. Both adolescents and their parents filled in a battery of questionnaires at home and during the lab visit, respectively.
